# Supplementary material for: MetaQTL: a package of new computational methods for the meta-analysis of QTL mapping experiments
Source: BMC Bioinformatics. 2007 Feb 8;8:49. doi: 10.1186/1471-2105-8-49 (PMC1808479; doi:10.1186/1471-2105-8-49)
Supplement: Additional File 7 — MetaQTL Package : jar file and tutorial. This Zip archive contains both the MetaQTL JAR file and the files of the tutorial. [file 1471-2105-8-49-S7.zip › org.inra.metaqtl/doc/org/metaqtl/IBioLocusProperties.html]

IBioLocusProperties


|  |  |  |
| --- | --- | --- |
| **Overview** \| **Package** \| **Class** \| **Use** \| **Tree** \| **Deprecated** \| **Index** \| **Help** | |  |
| **PREV CLASS**   **NEXT CLASS** | **FRAMES**    **NO FRAMES**     **All Classes** |  |
| SUMMARY: NESTED \| FIELD \| CONSTR \| METHOD | DETAIL: FIELD \| CONSTR \| METHOD |  |


---


## org.metaqtl Interface IBioLocusProperties

---

``` public interface IBioLocusProperties ```

This interface defines the key of the properties which
can be handled by a org.thalia.bio.entity.IBioLocus.

---

| **Field Summary** | |
| --- | --- |
| `static java.lang.String` | `DUBIOUS_ORDER`             The key for the marker dubious status. |
| `static java.lang.String` | `OCCURENCE`             The key for the occurence of markers in a consensus map |
| `static java.lang.String` | `QTL_CI_FROM`             The key for the QTL confidence interval lower boundary. |
| `static java.lang.String` | `QTL_CI_LOD_DECREASE`             The key for the QTL confidence interval LOD decrease. |
| `static java.lang.String` | `QTL_CI_TO`             THe key for the QTL confidence interval upper boundary. |
| `static java.lang.String` | `QTL_CROSS_NAME`             The key for the QTL cross name. |
| `static java.lang.String` | `QTL_CROSS_SIZE`             The key for the QTL cross size. |
| `static java.lang.String` | `QTL_CROSS_TYPE`             The key for the QTL cross type. |
| `static java.lang.String` | `QTL_PROJ_INTSCALE`             The key for the QTL flanking marker interval projection rate. |
| `static java.lang.String` | `QTL_PROJ_MAPSCALE`             The key for the QTL confidence interval projection rate. |
| `static java.lang.String` | `QTL_PROJ_ORIGIN`             The key for the map of origin of the QTL. |
| `static java.lang.String` | `QTL_PROJ_SHAREFLANKING`             The key for the QTL flanking marker interval order preservation. |
| `static java.lang.String` | `QTL_PROJ_SWAPFLANKING`             The key for the QTL flanking marker interval reverse order. |
| `static java.lang.String` | `QTL_STATS_ADDITIVE`             The key for the QTL additive effect. |
| `static java.lang.String` | `QTL_STATS_DOMINANCE`             The key for the QTL dominance effect. |
| `static java.lang.String` | `QTL_STATS_RSQUARE`             The key for the QTL rsquare. |
| `static java.lang.String` | `QTL_TRAIT_GROUP`             The key for the QTL trait group. |
| `static java.lang.String` | `QTL_TRAIT_NAME`             The key for the QTL trait name. |
| `static java.lang.String` | `QTL_TRAIT_UNIT`             The key for the QTL trait unit. |

**Field Detail**

### OCCURENCE

```
static final java.lang.String OCCURENCE
```

:   The key for the occurence of markers in a consensus map

    **See Also:**: Constant Field Values

---


### QTL\_TRAIT\_NAME

```
static final java.lang.String QTL_TRAIT_NAME
```

:   The key for the QTL trait name.

    **See Also:**: Constant Field Values

---


### QTL\_TRAIT\_UNIT

```
static final java.lang.String QTL_TRAIT_UNIT
```

:   The key for the QTL trait unit.

    **See Also:**: Constant Field Values

---


### QTL\_TRAIT\_GROUP

```
static final java.lang.String QTL_TRAIT_GROUP
```

:   The key for the QTL trait group.

    **See Also:**: Constant Field Values

---


### QTL\_CI\_LOD\_DECREASE

```
static final java.lang.String QTL_CI_LOD_DECREASE
```

:   The key for the QTL confidence interval LOD decrease.

    **See Also:**: Constant Field Values

---


### QTL\_CI\_FROM

```
static final java.lang.String QTL_CI_FROM
```

:   The key for the QTL confidence interval lower boundary.

    **See Also:**: Constant Field Values

---


### QTL\_CI\_TO

```
static final java.lang.String QTL_CI_TO
```

:   THe key for the QTL confidence interval upper boundary.

    **See Also:**: Constant Field Values

---


### QTL\_STATS\_RSQUARE

```
static final java.lang.String QTL_STATS_RSQUARE
```

:   The key for the QTL rsquare.

    **See Also:**: Constant Field Values

---


### QTL\_STATS\_ADDITIVE

```
static final java.lang.String QTL_STATS_ADDITIVE
```

:   The key for the QTL additive effect.

    **See Also:**: Constant Field Values

---


### QTL\_STATS\_DOMINANCE

```
static final java.lang.String QTL_STATS_DOMINANCE
```

:   The key for the QTL dominance effect.

    **See Also:**: Constant Field Values

---


### QTL\_CROSS\_NAME

```
static final java.lang.String QTL_CROSS_NAME
```

:   The key for the QTL cross name.

    **See Also:**: Constant Field Values

---


### QTL\_CROSS\_SIZE

```
static final java.lang.String QTL_CROSS_SIZE
```

:   The key for the QTL cross size.

    **See Also:**: Constant Field Values

---


### QTL\_CROSS\_TYPE

```
static final java.lang.String QTL_CROSS_TYPE
```

:   The key for the QTL cross type.

    **See Also:**: Constant Field Values

---


### DUBIOUS\_ORDER

```
static final java.lang.String DUBIOUS_ORDER
```

:   The key for the marker dubious status.

    **See Also:**: Constant Field Values

---


### QTL\_PROJ\_INTSCALE

```
static final java.lang.String QTL_PROJ_INTSCALE
```

:   The key for the QTL flanking marker interval projection
    rate.

    **See Also:**: Constant Field Values

---


### QTL\_PROJ\_MAPSCALE

```
static final java.lang.String QTL_PROJ_MAPSCALE
```

:   The key for the QTL confidence interval projection rate.

    **See Also:**: Constant Field Values

---


### QTL\_PROJ\_SHAREFLANKING

```
static final java.lang.String QTL_PROJ_SHAREFLANKING
```

:   The key for the QTL flanking marker interval order preservation.

    **See Also:**: Constant Field Values

---


### QTL\_PROJ\_SWAPFLANKING

```
static final java.lang.String QTL_PROJ_SWAPFLANKING
```

:   The key for the QTL flanking marker interval reverse order.

    **See Also:**: Constant Field Values

---


### QTL\_PROJ\_ORIGIN

```
static final java.lang.String QTL_PROJ_ORIGIN
```

:   The key for the map of origin of the QTL.

    **See Also:**: Constant Field Values


---


|  |  |  |
| --- | --- | --- |
| **Overview** \| **Package** \| **Class** \| **Use** \| **Tree** \| **Deprecated** \| **Index** \| **Help** | |  |
| **PREV CLASS**   **NEXT CLASS** | **FRAMES**    **NO FRAMES**     **All Classes** |  |
| SUMMARY: NESTED \| FIELD \| CONSTR \| METHOD | DETAIL: FIELD \| CONSTR \| METHOD |  |


---
